# Construction and accessibility of a cross-species phenotype ontology along with gene annotations for biomedical research

**DOI:** 10.12688/f1000research.2-30.v2

**Published:** 2014-01-21

**Authors:** Sebastian Köhler, Sandra C Doelken, Barbara J Ruef, Sebastian Bauer, Nicole Washington, Monte Westerfield, George Gkoutos, Paul Schofield, Damian Smedley, Suzanna E Lewis, Peter N Robinson, Christopher J Mungall

**Affiliations:** 1 Institute for Medical and Human Genetics, Charité-Universitatsmedizin Berlin, Berlin, 13353, Germany; 2 Berlin-Brandenberg Center for Regenerative Therapies (BCRT), Charité-Universitatsmedizin Berlin, Berlin, 13353, Germany; 3 ZFIN, Institute of Neuroscience, University of Oregon, Eugene OR, 97403-5291, USA; 4 Lawrence Berkeley National Laboratory, Berkeley CA, 94720, USA; 5 Department of Computer Science, University of Aberystwyth, Aberystwyth, SY23 2AX, UK; 6 Department of Genetics, University of Cambridge, Cambridge, CB2 3EH, UK; 7 Wellcome Trust Sanger Institute, Wellcome Trust Genome Campus, Cambridge, CB10 1SA, UK; 8 Max Planck Institute for Molecular Genetics, Berlin, 14195, Germany

## Abstract

Phenotype analyses, e.g. investigating metabolic processes, tissue formation, or organism behavior, are an important element of most biological and medical research activities. Biomedical researchers are making increased use of ontological standards and methods to capture the results of such analyses, with one focus being the comparison and analysis of phenotype information between species.

We have generated a cross-species phenotype ontology for human, mouse and zebrafish that contains classes from the Human Phenotype Ontology, Mammalian Phenotype Ontology, and generated classes for zebrafish phenotypes. We also provide up-to-date annotation data connecting human genes to phenotype classes from the generated ontology. We have included the data generation pipeline into our continuous integration system ensuring stable and up-to-date releases.

This article describes the data generation process and is intended to help interested researchers access both the phenotype annotation data and the associated cross-species phenotype ontology. The resource described here can be used in sophisticated semantic similarity and gene set enrichment analyses for phenotype data across species. The stable releases of this resource can be obtained from http://purl.obolibrary.org/obo/hp/uberpheno/.

## Introduction

Research on model organisms is crucial for discovering the function of genes and DNA elements and for understanding the phenotypic effects of mutations on these genes, which is leading to a better understanding of the pathobiology of human disease ^1, 2^. The amount of phenotypic information derived from targeted mutations and hypothesis-driven studies is increasing rapidly, and is now being further augmented by high-throughput international efforts to systematically analyse the effects of genomic variation on model organism phenotypes. For example, the International Mouse Phenotyping Consortium (IMPC ^3^), is undertaking systematic phenotyping studies of the knockouts generated by the International Knockout Mouse Consortium (IKMC ^4^). This means that there will soon be structured phenotype data for loss-of-function mutants for every protein-coding gene in the mouse. Similar approaches are being taken in zebrafish ( *Danio rerio*) by the Zebrafish Mutation Project (ZMP, http://www.sanger.ac.uk/Projects/D_rerio/zmp/) and the data is being made available through the Zebrafish Model Organism Database (ZFIN ^5^).

Model organism phenotype/genotype datasets are extremely valuable as they can provide clues to human gene functions and involvement in disease processes where no data is available for the human ortholog. At the time of writing, 2,358 human genes are associated with Mendelian phenotypes, but more importantly there are 5,492 human genes with no such phenotype associations, where an orthologous mouse or zebrafish gene does have phenotype data (Data obtained by analysing the file HSgenes_crossSpeciesPhenoAnnotation.txt from http://purl.obolibrary.org/obo/hp/uberpheno/). We have previously demonstrated the power of this approach in determining likely pathogenicity of genes within the intervals of recurrent copy number variation (CNV) diseases ^6^ and it can be applied much more widely in, for example, prioritizing candidate genes identified through human genome wide association studies (GWAS) ^7, 8^. Historically, a major problem has been the lack of common semantics across databases, with each project using some combination of free-text descriptions or in-house vocabularies. Thus, phenotype information is not easily integrated across different species. This inhibits comparisons based on phenotype alone, and where orthology is useful phenotypic comparisons cannot be used to their full potential. This is made even more complicated by different conceptualizations of phenotypes in different species and the impact of species-specific anatomies. As the ability of investigators to mobilise this growing collection of model organism data has become more important, it is crucial to develop appropriate ontologies and computational strategies to describe phenotypes such that phenotype descriptions can be objectively related to each other, both within and between species. This becomes even more important as the divergence between the number of human genes with phenotype information and the amount of systematically phenotyped model organism genes is expected to increase in the near future due to high throughput-screens ^1^.

The application of controlled vocabularies and ontologies has accelerated over recent years; the Gene Ontology (GO ^9^) being probably the most successful example in the field of biomedical ontologies. Many other ontologies exist, each of which has been developed for a specific domain in biomedicine. Now a major goal is to increase semantic and syntactic interoperability between those ontologies (e.g. the Open Biomedical Ontologies (OBO) Foundry ^10^). One approach is to develop ontologies by defining complex ("pre-composed") classes in terms of other more elementary (atomic) classes (building blocks) that are species-agnostic. If several ontologies make use of shared building block ontologies, interoperability can be facilitated across a larger domain. For example ontologies that contain classes concerned with *DNA-replication* in different organisms or cells should refer to a shared class representing *DNA-replication-process*, enabling computers to detect that the same class is referenced.

We have previously shown how phenotype information can be linked and used in cross-species phenotype analyses ^11– 15^. A crucial part of this strategy is the use of *logical definitions* to render ontology terms in a way that is computable. Recently, logical definitions of terms representing classes of phenotypic deviations have been developed by several groups. Developers of OBO Foundry ontologies, such as the GO ^16^, the Mammalian Phenotype Ontology (MPO ^17^), the Human Phenotype Ontology (HPO ^18, 19^), the Worm Phenotype Ontology ^20^, and also the Cell Ontology ^21^, are now creating logical definitions of their ontology-classes using terms from other building block ontologies. In this effort the Phenotype, Attribute and Trait Ontology (PATO), an ontology of phenotypic qualities, is a key tool ^19, 22^. Examples for building block ontologies that are used for the representation of classes of phenotypic abnormalities are given in the upper part of Table 1.

**Table 1.** Typical building block ontologies: here the focus lies on ontologies that can be used to represent complex classes of phenotype abnormalities in zebrafish, mouse, and human.

Domain	Name (Abbreviation, Reference)	Downloaded file (relative to http://purl.obolibrary.org/obo/)
biochemistry	Chemical Entities of Biological Interest (ChEBI ^29^)	chebi.obo
	Gene Ontology (GO ^30^)	go.obo
proteins	Protein Ontology (PRO ^31^)	pr.obo
cell types	Cell Ontology (CL ^32^)	cl.obo
anatomy	Foundational Model of Anatomy (FMA ^33^)	fma.obo
	Spatial Ontology (BSPO ^-^)	bspo.obo
	Mouse adult gross anatomy (MA ^34^)	ma.obo
	Zebrafish anatomy and development (ZFA ^35^)	zfa.ob
	Multi-species anatomy (UBERON ^36^)	uberon.obo
phenotype	Phenotype, Attribute and Trait Ontology (PATO ^22^)	pato.obo
	Mouse Pathology (MPATH ^37^)	mpath.obo
	Mammalian Phenotype Ontology (MPO ^17^)	mp.obo
	Human Phenotype Ontology (HPO ^18^)	hp.obo
	Neuro Behavior Ontology (NBO ^38^)	nbo.obo

## Objectives

Given that logical definitions exist for most classes of an ontology, automatic reasoners can be applied. These implement algorithms for computing the logical consequences that can be inferred from a set of asserted axioms. An example can be seen in Figure 1a), where logical definitions are used to automatically infer that *Hypoglycemia* is a subclass of *Decreased aldohexose concentration (blood)* based on the asserted subclass relationship between **'glucose'** and **'aldohexose'** in ChEBI. This means that reasoners are able to use computable, logical definitions to infer the positions of classes in a subsumption hierarchy. Thus, those definitions can be helpful tools for the development and maintenance of ontologies ^16, 23^.

**Figure:**
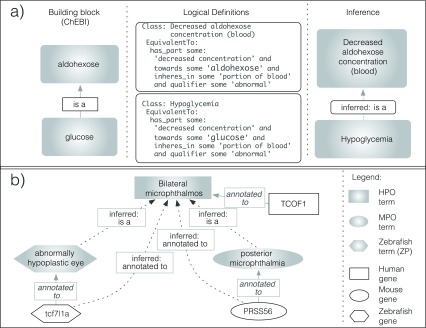
**Figure 1.** Part **a)** illustrates the main idea how logical definitions and building block ontologies (left) cooperate in order to allow for reasoning procedures to infer new knowledge (right). Note that for the purpose of increased readability, only the term labels are shown and the ontology Uniform Resource Identifier (URIs) are skipped. Part **b)** illustrates an excerpt of the *Uberpheno* ontology to show how information on phenotype abnormalities in different organisms can be combined. It also illustrates how the annotations of genes can be transferred across different species by means of orthology relationships of genes. For example, after reasoning one could easily request all genes that are known to be related to the phenotype description "Bilateral microphthalmos" from the HPO. In *Uberpheno* "abnormally hypoplastic eye" from zebrafish (ZP) and "posterior microphthalmia" from MPO, are inferred to be subclasses of "Bilateral microphthalmos". These inferences can be used to infer that the genes *tcf7l1a* (zebrafish) and *PRSS56* (mouse) are annotated to the phenotype "Bilateral microphthalmos" as well.

Although several methods, ideas, and applications on cross-species phenotype integration have been presented before ^11, 12, 16, 24, 25^, accessing such data resources has been complicated by the lack of consistent documentation and distribution of data across heterogenous resources. For example, some ontologies are provided in the Web Ontology Language (OWL ^26^) and others in the Open Biomedical Ontologies (OBO) format. Although the OBO-format focuses especially on human readability and ease of parsing, OWL is often needed to enable complex reasoning tasks. Unfortunately, the power and complexity of OWL may discourage some researchers.

For example, the OWLSim package ( http://owlsim.org) provides the ability to execute a number of standard semantic similarity techniques. Although access to the results of OWLSim in phenotype analyses is available ( ^25^, http://www.mousemodels.org), there is at the moment no single set of gene annotations linked to a single integrated ontology.

The *Uberpheno*-ontology is similar to the "phene.owl" ontology distributed as part of the phenomeblast-project ( http://code.google.eom/p/phenomeblast/) and generated as part of a phenotype data analysis executed within PhenomeNET ^24^. These two ontologies differ in a number of characteristics. The first characteristic is the underlying OWL model, and the set of external ontologies that are brought in to enrich the ontology - it is not yet clear how far the OWL model or some of these external ontologies affect the resulting structure of the ontology. Also it is likely that both *Uberpheno* and "phene.owl" will converge on the same model and a standard set of imported ontologies. The second characteristic is the breadth of species covered, with "phene.owl" including fly, worm and yeast; in contrast, *Uberpheno* focuses on human, mouse and zebrafish, yielding a smaller more focused ontology. Further investigations are required to determine the extent to which the adding of more distant organisms help or hinder analyses. Another difference is that *Uberpheno* is intended for a wide range of biomedical researchers, some of who may be unfamiliar with OWL or OWL reasoning.

Our objective here is to provide an OBO-format ontology ( *Uberpheno*), which we update at regular intervals and which can easily be used for downstream analysis, e.g. by applying semantic similarity measures ^27^ or gene set enrichment analyses ^28^. Of similar importance are the data that link into such an ontology by means of the annotation relation. To the best of our knowledge, no single integrated cross-species ontology together with annotation of all genes in human and model organisms (here mouse and zebrafish) has been made easily available for researchers and kept up-to-date on a regular basis.

## Materials and methods

### Model organism data

Cross-species ontology-based approaches offer a promising new methodology to reliably detect phenotypic similarities between human disease manifestations and model organism phenotypes ^6, 11, 24, 25^. They can pave the way to gain clinically relevant insights from the almost 5,500 genes for which, currently, only mouse and zebrafish phenotype information is available. Both the Mouse Genome Informatics (MGI) and the ZFIN data resources provide manually curated assignments of their model organism genes to human genes. They are available from the corresponding website (see Table 2).

**Table 2.** Files required to connect genes and phenotypes as well as to get the orthology relationship between model organism genes and human genes. These files are especially important for Step 4 in Figure 2.

Type	Organism	Obtain from
Orthology to human genes	Mouse	ftp://ftp.informatics.jax.org/pub/reports/HMD_HumanPhenotype.rpt
	Zebrafish	http://zfin.org/downloads/ortho.txt
Phenotype annotation	Mouse	ftp://ftp.informatics.jax.org/pub/reports/MGI_PhenoGenoMP.rpt
	Zebrafish	http://zfin.org/downloads/pheno.txt
	Human	http://compbio.charite.de/hudson/job/hpo.annotations/lastStableBuild/artifact/misc/phenotype_annotation.tab
Gene-to-disease	Human	<OMIM ftp-site>/ **mim2gene.txt** and <OMIM ftp-site>/ **genemap**
	Human	http://www.orphadata.org/data/xml/en_product6.xml

The annotation of genes to phenotypes are also accessible online. Zebrafish genes are annotated by Entity-Quality (EQ) statements. Mouse genes are annotated with terms from the MPO and are downloadable from the MGI website. To associate human genes with terms from the HPO, the annotation of human diseases is required. By using further files from OMIM ( http://omim.org) and Orphanet ^39^, ( http://www.orphadata.org/) diseases can be mapped to the disease-causing genes. These two steps allow the transfer of phenotype information to the underlying genes. All required files and their corresponding links are summarized in Table 2.

### Phenotype descriptions

The approach taken to logically define phenotype descriptions is termed the Entity-Quality approach (EQ), in which phenotype descriptions can be partitioned into (minimally) two parts. The first part represents the affected entity, i.e. the thing for which an observation is made. This can be entities of various domains, e.g., a chemical or an anatomical structure. The second part represents the quality of the entity and is described in a qualitative or quantitative way ^22^. In the typical setting, a phenotype is described using a class expression consisting of a PATO quality class differentiated by a bearer entity class using the **inheres_in** relation from the OBO Relation Ontology ^40^. To give an example for logical definitions, consider the HPO term *Hypoglycemia* and its EQ definition, specified in OWL as shown in Figure 1 (center).

The word *Hypoglycemia* refers to an abnormally decreased concentration of glucose in the blood. The logical definition uses relations and follows the pattern described in previous work on the definition of phenotypes ^16^. The logical semantics are made explicit when translating the definitions to OWL. Currently, the translation to OWL is performed using a **"has_part some"**-semantics implemented in the OBO-format library ( http://code.google.com/p/oboformat). The translation is shown in Manchester syntax in Figure 1a). In the example, the class *Hypoglycemia* is defined as the equivalent of the intersection of all classes of things that are "A concentration which is lower relative to the normal" ( *decreased concentration*), "deviate from the normal or average" ( *abnormal*), with respect to (towards) *glucose*, and inhering in "blood" (using the term *portion of blood* from the FMA). More details can be found in ^16^ or ^23^. Automated reasoning logically infers then that the asserted knowledge in ChEBI induces *Hypoglycemia* to be a subclass of *Decreased aldohexose concentration (blood)*. The files used to define phenotype classes are summarized in Table 3.

**Table 3.** Current statistics on the data contained in the used cross-product files. HPO and MPO files downloaded from http://code.google.com/p/phenotype-ontologies. Behaviour files downloaded from http://code.google.com/p/behavior-ontology. GO-xp file downloaded from http://obofoundry.org/cgi-bin/detail.cgi?id=biological_process_xp_uber_anatomy.

Ontology	File	Number of classes defined
HPO logical definitions	**hp-equivalence-axioms.obo**	4,666
MPO logical definitions	**mp-equivalence-axioms.obo**	7,278
GO logical definitions using Uberon	**biological_process_xp_uber_anatomy.obo**	1,484
Behavior xp	**behavior_xp.obo**	104

### *Uberpheno* construction

**Figure 2.**
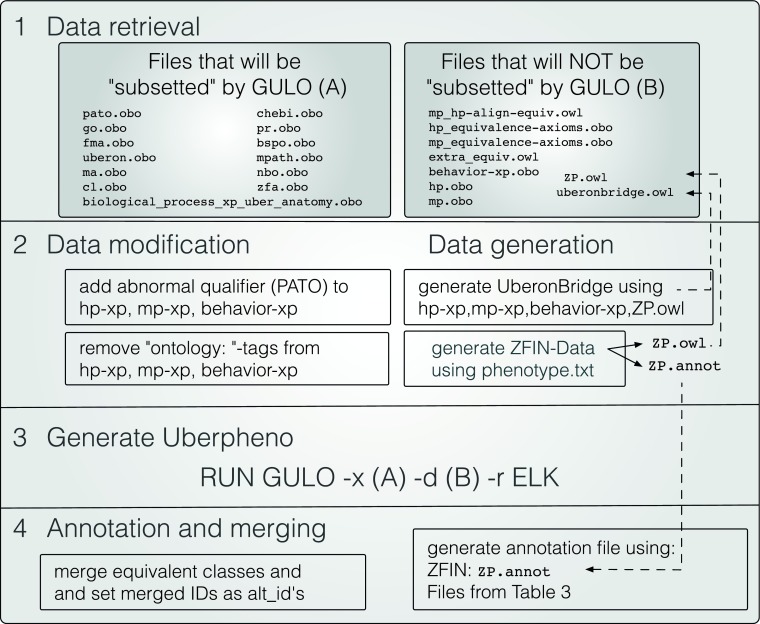
Schematic work- and dataflow illustration for the construction of the *Uberpheno* ontology and the gene annotations.

The general work- and data-flow of the cross-species ontology generation is illustrated in Figure 2. In steps one to three, the aforementioned EQ definitions are used to generate a single cross-species phenotype ontology ( *Uberpheno*) for human, mouse, and zebrafish phenotypes. Step four generates files that make it very convenient to use the generated data for several research purposes, because genes are linked to the terms of the generated cross-species phenotype ontology, which is very lightweight and available in the convenient OBO-format.

***Step 1***. Logical definitions are being developed for GO ^16^, MPO ^12^, and HPO ^19^. Almost all logical definitions refer to classes from other ontologies. A set of logical definitions is again an ontology itself. These bridging ontologies (also called cross-product files) are available on the main OBO Foundry website, as well as from the individual repositories for each of the projects. An example for a logical definition is presented in the previous section and in Figure 1. A major fraction of HPO and MPO terms are currently defined by means of EQ statements and a summary of the logical definition files that are used can be found in Table 3. These files provide axioms that connect phenotype classes to multiple classes in most of the ontologies listed in Table 1.

The HPO and MPO logical definitions were augmented with pairwise equivalence axioms generated by lexical matching. These mappings are represented in a file **mp_hp-align-equiv.owl** (see the phenotype ontologies archive on Google code at http://code.google.com/p/phenotype-ontologies). A total of 1,064 such lexically derived equivalence axioms were derived in this way and used to supplement the semantic analysis.

In step one, all of the required files are pulled from the web (see Table 1 and Table 3). Note, that there are ontologies that are required in their entirety (denoted (B) in Figure 2). In contrast, several building block ontologies (denoted (A) in Figure 2) are only referred in parts by the logical definitions.

When defining phenotypes using the EQ model, the affected entity can either be a biological function or process from GO, or an anatomical entity. Some of the ontologies used to create the definitions are largely species-independent (GO, ChEBI). However, anatomical entities are mostly defined by referring anatomy ontologies that are specific for one species. In order to enable reasoning across these vertebrate anatomies, the metazoan, species-independent Uberon ontology is used in constructing anatomically-based cross-products ^36^. In order to construct *Uberpheno*, an equivalence axiom was generated between every class in Uberon that contains a cross-reference to a species anatomy ontology class. Note that very general terms from Uberon such as *tissue* are excluded, which can be identified by their membership to the subset **upper_level** in Uberon. The generated file is called **uberonbridge.owl**.

One of the files (see Table 3) defines GO process terms by the anatomy term to which the process is related. For example,

**Class: eye pigmentation**

**EquivalentTo:**

**pigmentation and**

**occurs_in some eye**

Here, the GO process *eye pigmentation* ( GO:0048069) is logically defined as being equivalent to everything that is a *pigmentation* ( GO:0043473) and also **"occurs_in"** an *eye* ( UBERON:0000970). In order to use these definitions, the different relationships used therein, such as **occurs_in**, are made interpretable for the reasoner. For our purposes, an additional ontology called http://compbio.charite.de/svn/hpo/trunk/misc/go_xp_misc/extra_equiv.owl was created in which these relationships are made a **subPropertyOf** of **inheres_in**.

***Step 2***. In step two a data preprocessing is required, because for zebrafish no pre-composed ontology of phenotype abnormalities exists (e.g. no phenotype term such as *abnormally hypoplastic eye* exists). Instead, the ZFIN project makes use of so-called "post-composed" annotations, using a combination of classes in the EQ model. The ZFIN-file **pheno.txt** ( Table 2) contains lines such as

**ZDB-GENE-980605-30;83439;tcf7l1a;ZFA:0000107;eye;PATO:0000645;hypoplastic;abnormal**

For legibility the tab-separators are replaced in this example by the semicolon. In order to use these annotations for reasoning, a translation table was implemented, as described before ^12^, which generates the ontology denoted as **zp.owl**. For every modified gene, a set of post-composed phenotype annotations is stored in **pheno.txt**. For every unique annotation for zebrafish genes, a class in the ZP identifier space is created. Again, the aforementioned **"has_part some"**-translation to OWL is applied. For example, a zebrafish gene annotation with

**Entity=ZFA:0000107 (eye),**

**Quality=PATO:0000645 (hypoplastic) and**

**Qualifier=PATO:0000460 (abnormal)**

generates an OWL class:

**Class: ZP_0003395**

**Annotations: label "abnormal(ly) hypoplastic eye"**

**EquivalentClassOf:**

**has_part some:**

**PATO_0000645 and**

**inheres_in some ZFA_0000107 and**

**qualifier some PATO_0000460**

Beside generating the ZP-ontology, the annotation relation between the zebrafish genes and ZP-term is written to a file called **zp.annot**, which is also available for download.

Since some logical definitions of phenotypes are lacking the qualifier *abnormal* we ensure consistency, by adding this qualifier to all of the definitions. We also remove the inconsistently used ontology-tags from the xp-files.

***Steps 3 and 4.*** At first, a single, merged OWL ontology is created from all the ontologies and bridging axioms. The ELK reasoner ^41^ is used to calculate subclass and equivalence relationships between classes. These steps are implemented within the GULO framework ^23^.

To increase the usability of the ontology, the Ontologizer API ^28^ was used to merge all clusters of equivalent classes together into a single class. The HPO identifier is taken as the primary identifier if present and the identifiers of other phenotype classes are stored under **alt_id**-tag for the term. For example, the HPO-term *Gallbladder dysfunction* ( HP:0005609) has as **alt_id** the ZP-term *abnormal(ly) decreased functionality gall bladder (ZP:0004170)*. The resulting ontology in OBO-format is named **crossSpeciesPheno.obo** and contains only phenotype classes from the HPO, MPO, and ZP.

Finally a cross-species annotation file is generated, in which all human genes are associated with terms from the *Uberpheno*. The annotations are either stemming from human or model organisms, whereby the model organism annotations are stemming from the ortholog gene.

## Results and discussion

All of the above described methods are integrated into a single pipeline. This pipeline automatically downloads required files, preprocesses the data and applies a reasoning procedure to the obtained set of ontology classes. The ontologies used to construct *Uberpheno* are summarized in Table 1.

The construction pipeline is set up as a job in our continuous integration system accessible at http://compbio.charite.de/hudson, which is already used for data related to the HPO ^42^. The job (called **hpo.ontology.uberpheno**) is configured to run once a week, ensuring that the most recent version of all ontologies and annotation files are used. Only stable releases of the generated files are made available to the users and errors are immediately forwarded to us via email. The generated build artifacts are available at http://purl.obolibrary.org/obo/hp/uberpheno/, whereas the file **crossSpeciesPheno.obo** contains the cross-species phenotype ontology in OBO-format. The resulting ontology has a light footprint (3.5 MB) and can easily be explored by using tools such as example OBO-Edit ^43^. Note that only phenotype classes are present in the ontology and classes from the referenced building block ontologies are filtered out. Each build also generates the file **HSgenes_crossSpeciesPhenoAnnotation.txt**, which contains the annotation of all human genes to terms of HPO, MPO, and ZP. A summary of the data contained in the two files is given in Table 4.

**Table 4.** Statistics of the build artifacts generated (build #63). 'Phenotype classes' denotes the number of classes that are either from the Human Phenotype Ontology (HPO), Mammalian Phenotye Ontology (MPO), or **zp.owl** (ZP). Note that the sum of HPO-, MPO-, and ZP-IDs is higher than the total number total 'Phenotype classes' because some MPO- and ZP-IDs are listed as **alt_id** of an HPO-class and are not listed as separate 'Phenotype class'. Also, the number of human annotations is less than the sum of annotations supported by OMIM or Orphanet entries, because some annotations have evidence from both databases.

*Statistics*	
*Uberpheno statistics:* Phenotype classes: HPO-IDs MPO-IDs ZP-IDs	25,974 13,122 9,800 8,057
*Annotation statistics:* All annotations HPO annotations -OMIM -Orphanet MPO annotations ZP annotations	235,752 63,080 49,348 16,244 149,164 23,508

An excerpt of the *Uberpheno* ontology is shown in ( Figure 1b), demonstrating how the phenotype descriptions from different ontologies are combined and automatically organised into a single, integrated hierarchy. For instance, the fact that the mouse term *posterior microphthalmia* is inferred to be a subclass of the human term *Bilateral microphthalmos* can be used to transfer the information that the mouse gene *PRSS56* is known to cause *Bilateral microphthalmos*. This implies that querying the cross-species ontology for genes related to *Bilateral microphthalmos* will return the human gene *TCOF1*, the mouse gene *PRSS56* and the zebrafish gene *tcf7l1a*.

In total, the annotation file contains approx. 235,000 annotations of human genes with phenotype classes (see Table 4). For example the human gene *TCF7L1* is associated with the zebrafish phenotype *abnormal(ly) hypoplastic eye* because the ortholog zebrafish gene ( *tcf7l1a*, ZDB-GENE-980605-30) is annotated with this phenotype. Thus, the generated file **HSgenes_crossSpeciesPhenoAnnotation.txt** contains the line:

**83439;TCF7L1;abnormal(ly) hypoplastic eye (ZP:0003395);tcf7l1a (ZDB-GENE-980605-30/ZEBRAF)**

## Conclusions

The phenotype resources for mouse, zebrafish, and human are used by several research projects ^44– 46^.

The problem of comparing phenotypes between species can be overcome by using formal logical definitions that make use of species agnostic ontologies together with a multi-species anatomy ontology, Uberon. The approach to implementing the paradigm that we report in this paper constructs a single, integrated, cross-species phenotype ontology, *Uberpheno*, based on the logical definitions of human and the main model species, mouse and zebrafish. The resulting construct is continuously updated and automatically constructed as the constituent ontologies are updated and augmented, making it a dynamic and current resource available to the community.

Increasingly model organism data are being used for gene set enrichment, pathogenicity prediction and semantic similarity analyses ^27^ and the high throughput phenotyping projects newly underway promise rich genome-wide phenotypic coverage within a decade. This will complement the new initiatives to systematically gather high precision, formally coded, phenotype data from clinical studies ^47^. The promise that all this data holds can only be realized if the informatics tools are available to handle and analyse this rich resource and we believe that *Uberpheno* is an accessible and widely applicable resource with which this may be achieved.
