# Supplementary material for: Construction and accessibility of a cross-species phenotype ontology along with gene annotations for biomedical research
Source: F1000Res. 2014 Jan 21;2:30. Originally published 2013 Feb 1. [Version 2] doi: 10.12688/f1000research.2-30.v2 (PMC3799545; doi:10.12688/f1000research.2-30.v2)
Supplement: Peer review history

# Peer review: Referee response for version 2

**DOI:** 10.5256/f1000research.3649.r4261

**Published:** 2014-03-27

**Authors:** Larry Hunter

**Affiliations:** 1 Center for Computational Pharmacology & Computational Bioscience Program, UC Denver, Aurora, CO, USA

I have read this submission. I believe that I have an appropriate level of expertise to confirm that it is of an acceptable scientific standard.

# Peer review: Referee response for version 2

**DOI:** 10.5256/f1000research.3649.r3279

**Published:** 2014-01-23

**Authors:** Nicola Mulder

**Affiliations:** 1 Computational Biology Group, Institute of Infectious Disease and Molecular Medicine, Cape Town, South Africa

I have read this submission. I believe that I have an appropriate level of expertise to confirm that it is of an acceptable scientific standard.

# Peer review: Referee response for version 2

**DOI:** 10.5256/f1000research.3649.r3271

**Published:** 2014-01-22

**Authors:** Francisco Couto

**Affiliations:** 1 Large-Scale Informatics Systems Laboratory (LASIGE) Lisbon, University of Lisbon, Campo Grande, Lisbon, Portugal

I have read this submission. I believe that I have an appropriate level of expertise to confirm that it is of an acceptable scientific standard.

# Peer review: Referee response for version 1

**DOI:** 10.5256/f1000research.905.r764

**Published:** 2013-02-12

**Authors:** Nicola Mulder

**Affiliations:** 1 Computational Biology Group, Institute of Infectious Disease and Molecular Medicine, Cape Town, South Africa

This paper describes a cross-species phenotype ontology, which promises to be extremely useful. It was not clear to me how much of the data preparation was purely computational versus some biological input. One has to be wary of trying to make too many terms apply to multiple species, but given the collective experience of these authors I am sure this has been taken into consideration.

I have read this submission. I believe that I have an appropriate level of expertise to confirm that it is of an acceptable scientific standard.

# Peer review: Referee response for version 1

**DOI:** 10.5256/f1000research.905.r761

**Published:** 2013-02-11

**Authors:** Francisco Couto

**Affiliations:** 1 Large-Scale Informatics Systems Laboratory (LASIGE) Lisbon, University of Lisbon, Campo Grande, Lisbon, Portugal

The availability of a cross-species phenotype ontology built as a mashup of different other ontologies is a great contribution to the community and results from the implementation of a well-designed integration process. The process combines knowledge from different sources taking in account their provenance, which guarantees the continuous update of the proposed ontology. The successful exploitation of logical definitions is also a very good contribution to promoting the usage of more formal definitions which I believe will, for example, help to enhance the reliability of semantic similarity and enrichment analyses.

I have read this submission. I believe that I have an appropriate level of expertise to confirm that it is of an acceptable scientific standard.

# Peer review: Referee response for version 1

**DOI:** 10.5256/f1000research.905.r757

**Published:** 2013-02-08

**Authors:** Larry Hunter

**Affiliations:** 1 Center for Computational Pharmacology & Computational Bioscience Program, UC Denver, Aurora, CO, USA

This is really nice work, clearly demonstrating the value of logical definitions of ontology terms and of inference over those definitions. The automatic updating of the inference, ensuring that UberPheno reflects current annotations (and definitions, which change less frequently) should be adopted as a 'best practice' by other providers of derived information.

I have read this submission. I believe that I have an appropriate level of expertise to confirm that it is of an acceptable scientific standard.
